# Generation of polychromatic projection for dedicated breast computed tomography simulation using anthropomorphic numerical phantom

**DOI:** 10.1371/journal.pone.0187242

**Published:** 2017-11-06

**Authors:** Hosang Jeon, Hanbean Youn, Jin Sung Kim, Jiho Nam, Jayoung Lee, Juhye Lee, Dahl Park, Wontaek Kim, Yongkan Ki, Donghyun Kim

**Affiliations:** 1 Department of Radiation Oncology and Research Institute for Convergence of Biomedical Science and Technology, Pusan National University Yangsan Hospital, Gyeongsangnam-do, South Korea; 2 Department of Radiation Oncology, Yonsei Cancer Center, Yonsei University College of Medicine, Seoul, South Korea; 3 Department of Radiation Oncology, Pusan National University Hospital, Busan, South Korea; University of California Davis, UNITED STATES

## Abstract

Numerical simulations are fundamental to the development of medical imaging systems because they can save time and effort in research and development. In this study, we developed a method of creating the virtual projection images that are necessary to study dedicated breast computed tomography (BCT) systems. Anthropomorphic software breast phantoms of the conventional compression type were synthesized and redesigned to meet the requirements of dedicated BCT systems. The internal structure of the breast was randomly constructed to develop the proposed phantom, enabling the internal structure of a naturally distributed real breast to be simulated. When using the existing monochromatic photon incidence assumption for projection-image generation, it is not possible to simulate various artifacts caused by the X-ray spectrum, such as the beam hardening effect. Consequently, the system performance could be overestimated. Therefore, we considered the polychromatic spectrum in the projection image generation process and verified the results. The proposed method is expected to be useful for the development and optimization of BCT systems.

## Introduction

Dedicated breast computed tomography (BCT) is one of the emerging imaging techniques for diagnosing breast cancer. It has been extensively studied in recent years to overcome the disadvantages of existing breast cancer diagnostic methods, especially the inconveniences caused by compression and the inability to acquire 3D images [1–7]. Typically, numerical simulations are essential in medical imaging studies to obtain preliminary results. The most widely used and most accurate method of simulating image systems is the Monte Carlo method. However, the Monte Carlo simulation accuracy increases in proportion to the number of histories employed, which is disadvantageous in terms of computational complexity. The forward-projection-based simulation method is a suitable and easy alternative and is used in various fields. However, this method is disadvantageous in that it does not sufficiently replicate actual scenarios, because phantoms with simple geometries are generally used and the X-ray spectra are ignored by making monochromatic assumptions.

Software phantoms mimicking imaging objects, which are typically combinations of simple geometrical shapes (e.g., spheres, ellipsoids, and cubes), are essential to implement numerical simulations. Owing to their simplistic structure, however, more realistic and/or complex software phantoms have been suggested. For breast imaging, there are two primary types of software phantoms: 1) voxel-based software breast phantoms randomly generated using the region-growing algorithm [8–10] and 2) software breast phantoms replicated from actual volumetric image sets through magnetic resonance imaging or computed tomography [11–13]. Phantoms of the former type have realistic anatomical geometries and are only focused on the compressed breast for digital breast mammography and tomosynthesis. Those of the latter type can provide uncompressed software breast phantoms with gravitational force, which are suitable for numerical BCT simulations. However, the limited spatial resolutions of actual clinical image sets (~1 mm) may hamper the generation of precise software phantoms. Moreover, the accuracies of the voxel densities of the generated phantoms cannot be verified.

Projection-image generation is commonly used in ray- [14], pixel- [15], and distance-driven [16, 17] algorithms and their speed-improved versions [18–20]. However, significant computational effort is required to obtain high-resolution simulation images. Therefore, it is common to use the monoenergetic approximation because it is difficult to perform practical simulations considering the entire polychromatic spectrum. However, by assuming a monoenergetic spectrum, artifacts and errors caused by the polyenergetic spectrum, such as the Swank noise factor [21] and beam hardening effect [22], cannot be simulated. To solve this problem practically, acceleration methods involving the use of a graphics processing unit (GPU) [23] or undersampling of the incident spectrum itself [24, 25] have been suggested. Nevertheless, these methods are disadvantageous in that they can distort multi-peak spectra and require additional hardware, such as a GPU, and high-level programming skills.

In this report, we propose a non-compressed breast-specific software phantom and projection-image generation process considering polyenergetic X-ray spectra purely through computer simulations. We describe the algorithm for breast phantom development through random number generation and the projection image generation process considering the polychromatic spectrum. We expect that these techniques will enable simulation of the image quality, the upper bound of which is determined by the system parameters. To overcome the disadvantages of the conventional forward-projection-based method, we propose a more realistic phantom and a projection-image generation algorithm developed by considering the polychromatic X-ray spectrum.

## Methods

### Breast anatomy

The human female breast is one of the most complex organs to be imaged and visually inspected because of its complex internal structure and the similar tissue densities of its components. The components of the breast can be categorized as functional and surrounding units. The functional units include the ductal networks and lobular acini that are involved in breast milk production. The surrounding tissue units include the adipose and connective tissue, which maintain the overall shape of the breast, as well as the enclosing ducts and lobules. The X-ray attenuation coefficients of the functional and surrounding units, except for fat, are almost identical and randomly mixed; hence, the breast is the most challenging area to image using X-ray imaging.

Anatomically, each breast has seven to nine lobules that branch off from the nipple. Each lobe contains a ductal network and lobules at the ends of the ducts. They are the most important structures for producing and transporting milk to the alveoli, and tumors are most likely to occur in this region. The number of ducts in the breast is commonly known to be 15–20. The numbers of lobules and terminal ducts are approximately 20–40 and 10–100, respectively. Since the ductal network is randomly divided into acute angles, it is distributed along the nipple to the chest wall. The ligaments that maintain the external shape are divided into fat cells at the border of the skin and lineage. Based on this anatomical information, we applied constraints to model each breast component and the limiting random variables. The details of the anatomical constraints are listed in Table 1.

**Table 1:** Anatomical distributions of breast components. All of the distributions were assumed to be Gaussian.

Category	Units	Mean	Standard deviation
External shape (diameter)	mm	140	20
External shape (thickness)	mm	120	10
Skin	mm	5	0
Number of ducts starting	-	17	3
Number of lobes	-	17	3
Number of lobules per lobe	-	30	10
Number of terminal ducts per lobe	-	50	50

### Design of anthropomorphic breast phantom

To develop a natural and randomized anthropomorphic breast phantom, we modified the random generation model described by Bliznakova [10]. In that model, random numbers are used to generate a cylindrical ductal network. The breast tissue distribution simply fills a noisy array, which has continuous values with Fourier filtering to express the internal structure of congested breasts. However, to generate polychromatic projections, the distribution inside the phantom should be characterized using integers to represent the component index. To model the normal structure, we first divided the breast anatomy into ductal networks, lobules, and Cooper’s ligaments.

#### External boundary and skin layer

**Fig 1:**
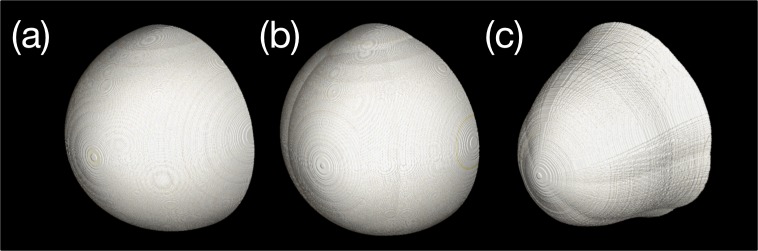
Examples of external boundary generation: (a) single-hemisphere model, (b) multiple-hemisphere model, and (c) individual plane combined model. The creases observable in (c) were randomly generated at the overlap between the circles used to model the axial slice. This phenomenon can be reduced by increasing the number of circles. However, because the external boundary of the breast is not a critical part, it was ignored in this study.

The external shape of a breast covered with a skin layer can be modeled as a hemisphere, as shown in Fig 1(A). This model can be made more complex by merging multiple hemispheres, as depicted in Fig 1(B). In fact, these simple shapes are sufficient to design breast phantoms because the external shape does not significantly affect the phantom quality. However, we used random numbers to generate the appearance of the breast in the prone position to create a more visually realistic phantom. The generation of such a detailed external shape could be useful because it can serve as a guideline for roughly determining the sizes of detectors in BCT systems.

**Fig 2:**
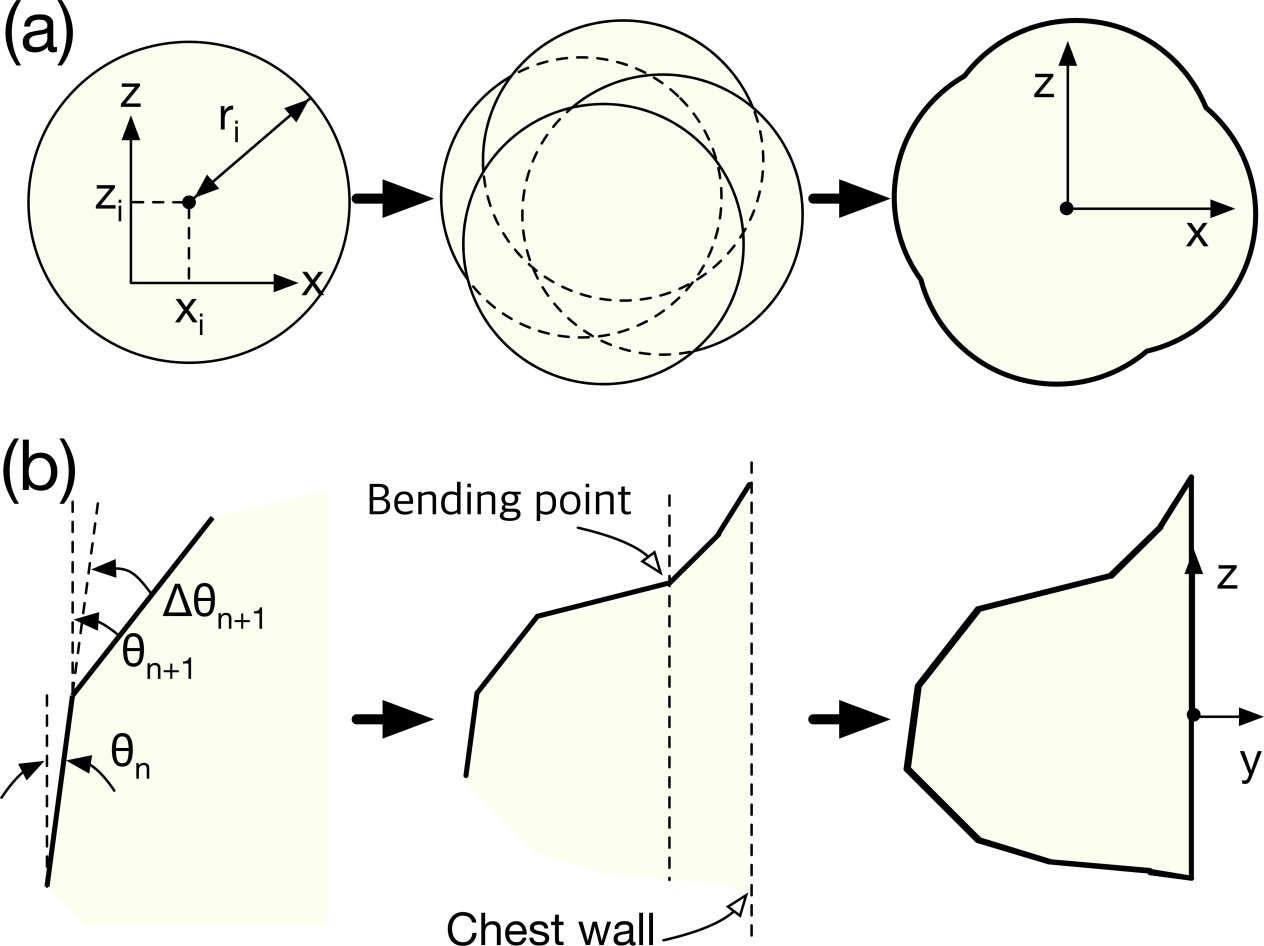
Generation of the external boundary of the breast phantom: (a) axial (*x*-*z*) and (b) sagittal (*y*-*z*) planes.

To increase the level of detail, the external boundary was modeled using a combination of the axial (*x-y*) and sagittal (*y-z*) planes. The axial plane at the bottom of the breast was created from a combination of several circles, as shown in Fig 2(A), and the positions and diameters of the circles were randomized. Since the mean and standard deviation of the diameter of the nominal breast were 90 mm and 20 mm, respectively, as indicated in Table 1, we determined the centers of the circles (*x*_*i*_, *z*_*i*_) using

$$\left\{ \begin{array}{c} x_{i}=\mathrm{rand}(-1,1)\times 10\\ z_{i}=\mathrm{rand}(-1,1)\times 10 \end{array}\right.,$$ (1)

where r and(-1,1) is a random number generator following the normal distribution in the range [–1, 1]. Similarly, we denote a random number generator bounded between *a* and *b* as rand(*a*,*b*). The subscript *i* denotes the number of circles. Empirically, the appropriate number of circles is 3 or 4. As the number of circles increases, the shape of the axial plane becomes increasingly irregular.

The sagittal plane was simulated from the position of the nipple. The shapes of the top and bottom relative to the nipple differed, as shown in Fig 2(B). The basic method involved modeling the curvature of the sagittal plane as a combination of straight lines. The bending angle *θ*_*n+*1_ for the (*n*+1)-th straight line was randomly assigned to be the positive direction. The differential angle between *θ*_*n*_ and *θ*_*n+*1_ was empirically bounded from 0° to 10°, enabling *θ*_*n+*1_ to be determined using

$$\theta_{n+1}=\theta_{n}+rand(0,1)\times 10^{\circ }.$$ (2)

To model the deflection caused by the chest-wall connection, the curvature was specified in the negative direction from a point located at a depth of one-fourth the total breast depth. Therefore, *θ*_*n+*1_ as measured from the bending point, which is shown in Fig 2(B), could be expressed as

$$\theta_{n+1}=\theta_{n}-rand(0,1)\times 10^{\circ }.$$ (3)

The simulation of the bottom part of the sagittal plane was in the direction opposite to that of the top part. However, the angular inversion at the bending point caused by the chest wall was ignored.

Finally, the external boundary of the breast was obtained by combining the sagittal and axial planes. The three dimensions were combined through slice-by-slice mapping by resizing the diameter of the axial plane to the thickness of each slice of the sagittal plane. Since the proposed phantom is a voxel phantom, the mapping interval of each slice is equal to the slice thickness.

#### Ductal network

Since the ductal network model follows that proposed by Bliznakova, this section only provides a brief introduction and focuses on the differences between our model and that developed by Bliznakova. To generate the ductal network, we assumed it to be a combination of cylinders. When generating a network, three constraints are necessary to maintain realism: 1) due to milk flow conservation, the cross-sections of the cylinders must be preserved before and after branching; 2) a cylinder can be divided into two branches only; 3) the cylinders must only branch by acute angles. Using these constraints, we numerically simulated a ductal network system.

The random numbers used in this procedure are the branching angle (azimuthal and polar), the length of the unit cylinder, and a random variable that determines branching. At the end of a unit cylinder, which has a random direction and length, we decided to create a random split. At that time, we designated the initial branch and split branches as “mother” and “child” branches, respectively. Each child branch became a mother branch for subsequent child branches. The random values of the ducts are provided in Table 2.

**Table 2:** Parameters utilized to construct the ductal network.

Category	Parameter	Units	Mean value
Major duct	Length	mm	1
	Radius	mm	2
Lactiferous ducts	Length	mm	7
	Radius	mm	1
	Polar angle	degrees	30
	Azimuthal angle	degrees	60

In the method proposed by Bliznakova, each cylinder is assumed to have the shape of a cropped cone and different initial and terminal diameters are defined. That is, the diameter of the ductal network decreases continuously from the nipple to the chest wall. However, considering the milk flow conservation mentioned above, this assumption could be excessive; therefore, we replaced the cone with a simple cylinder. The diameter changes of the cylinders were achieved by branching each cylinder into two while preserving the sum of the cross-sectional areas. During branch generation, the diameters of the two cylinders are not evenly spaced. We used an additional random number to specify the diameters of the divided branches (*d*_*d*,1_, *d*_*d*,2_) as

$$\left\{ \begin{array}{c} d_{d,1}=2\sqrt{a_{0}\frac{d_{cyl}}{\pi }}\\ d_{d,1}=2\sqrt{a_{0}\frac{1-d_{cyl}}{\pi }} \end{array}\right.,$$ (4)

where *d*_*cyl*_ is the randomly assigned cylinder diameter, which can be obtained using r and (0,1). The branch angles and lengths should be slightly adjusted due to the external boundary of the phantom to prevent skin invasion. If child branches are not generated, then differential azimuthal and polar angles are re-assigned only to redirect the mother branch. Based on the differential angles, the cylinder will grow along the altered direction with a constant diameter. On the other hand, if child branches are generated, two differential angles are assigned in the positive and negative directions with respect to the perpendicular plane of the mother branch. The growth algorithm is applied in the same manner for both directions.

Since the lobular acini form one of the main components of glandular tissue, the ductal-network distribution determines the distribution of glandular tissue within the breast phantom. Lobular acini are mainly located at the distal end of the ductal network. Therefore, when the cylinder constituting the terminal duct is generated, the corresponding position is recorded separately. This process is applied to the glandular tissue distribution model explained in the next subsection.

#### Cooper’s ligament and glandular tissue distribution

Cooper’s ligament consists of connective tissue that maintains the global structure of the breast. To express the connectivity of Cooper’s ligament, it can be characterized as a group of vacant cells having a thin membrane. Modeling of the ligament membrane can be begun by constructing a unit hollow volume with a thin surface.

Although the hollow volume can simply be an ellipsoid, we applied random number generation to attain more reliable complexity. The *i*-th ellipsoid of a hollow membrane is given by

$$\frac{(x-x_{C,i}{)}^{2}}{a_{C,i}^{2}}+\frac{(y-y_{C,i}{)}^{2}}{b_{C,i}^{2}}+\frac{(z-z_{C,i}{)}^{2}}{c_{C,i}^{2}}=1.$$ (5)

All of the variables could be random. The random variables for the *i*-th ellipsoid for a ligament group are the lengths of each axis (*a*_*C*,*i*_, *b*_*C*,*i*_, *c*_*C*,*i*_), rotation along each axis (*θ*_*C*,*x*_, *θ*_*C*,*y*_, *θ*_*C*,*z*_), and centers of the ellipsoids (*x*_*C*,*i*_, *y*_*C*,*i*_, *z*_*C*,*i*_). Table 3 summarizes the random parameters obtained empirically. Each mean value is combined with a random number generator, r and (0, 2).

**Table 3:** Parameter values used to define Cooper’s ligament.

Parameters	Units	Mean value
*x_C,i_, y_C,i_, z_C,i_*	mm	5
*θ_C,x_, θ_C,y_, θ_C,z_*	degrees	360
***a_C,i_, b_C,i,_,*** *c****_C,i_***	mm	20

**Fig 3:**
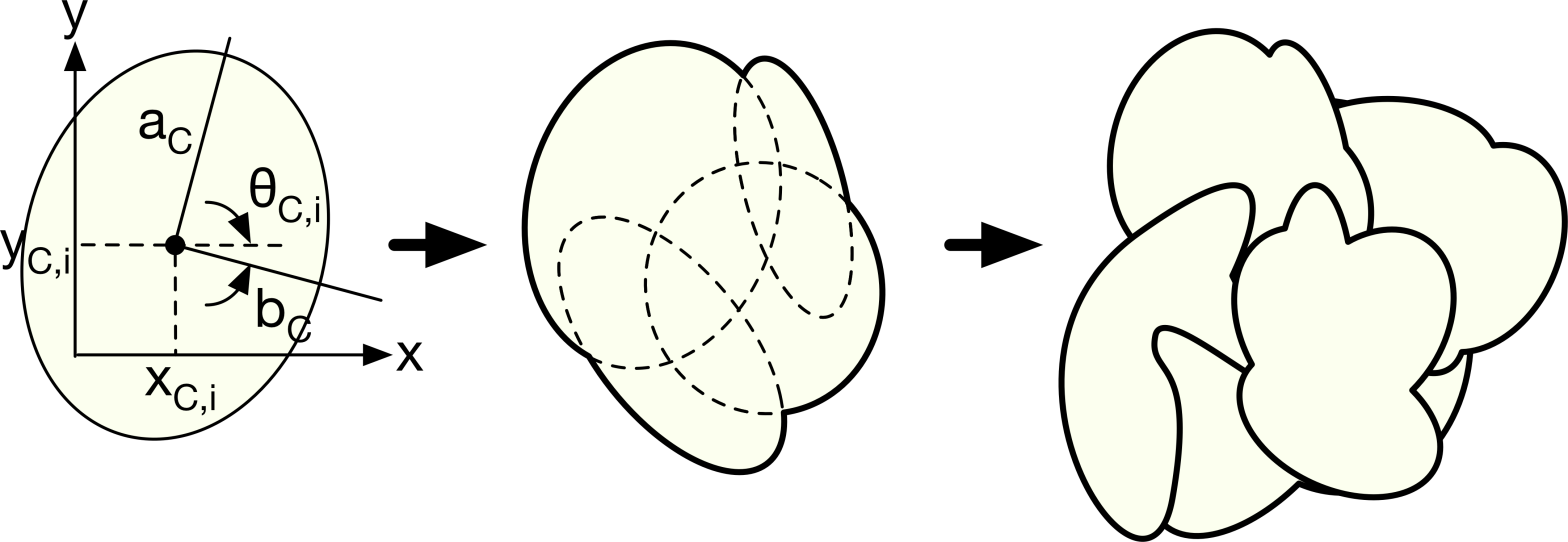
2D simplification of Cooper’s ligament generation model. Random ellipsoids are first constructed using random parameters; then, a random unit ligament is generated using “AND” operations. The same operation is repeated until the interior of the phantom is completely filled.

A unit of Cooper’s ligament is generated using the union (or “OR” operation) of several generated ellipsoids. Unit generation is continued until the inside of the breast is filled with Cooper’s ligament. To facilitate understanding, the ligament generation model is schematically illustrated in two dimensions in Fig 3. The inner part of the membrane of a unit of Cooper’s ligament is used to limit the distribution of lobular tissue. Since the lobule distribution can be anatomically exaggerated, it is suitable to limit the distribution around the terminal duct.

Lobular tissue (or lobules) is made of lobular acini, which are macroscopically non-arranged cell clusters. Lobular tissue, therefore, cannot be shaped using standardized patterns, because it changes beyond the sub-voxel size. To overcome this issue, we extracted lobular tissue from the normally distributed white noise array in three dimensions by Fourier filtering of the 3D array containing white noise. It is known that the frequency filter function *W* describing the glandular tissue distribution obeys the following inverse power law in the frequency domain [26, 27]:

$$W(f)=\frac{\alpha }{f^{\beta }},$$ (6)

where α and *β* are the magnitude and exponent variables in terms of spatial frequency *f*, which determine the breast density. The default values of α and *β* were set to 1 and 2, respectively, in this study. *β* can be adjusted to acquire a certain distribution intentionally. A higher *β* produces a smoother distribution. We assumed the frequency distribution to be rotationally symmetric; hence, *f* is the frequency in the radial direction. Based on the frequency-filtered noise array *N*_*f*_, thresholding-based binarization was applied to separate glandular and adipose regions, enabling the adipose and glandular distribution *N*_*b*_ to be expressed as

$$N_{b}(x,y,z)=\left\{ \begin{array}{c} 1,\;\mathrm{where}\;N_{f}(x,y,z)\geq t\;(\mathrm{glandular}\;\mathrm{tissue})\\ 0,\;\mathrm{where}\;N_{f}(x,y,z)<t\;(\mathrm{adipose}\;\mathrm{tissue}) \end{array}\right..$$ (7)

**Fig 4:**
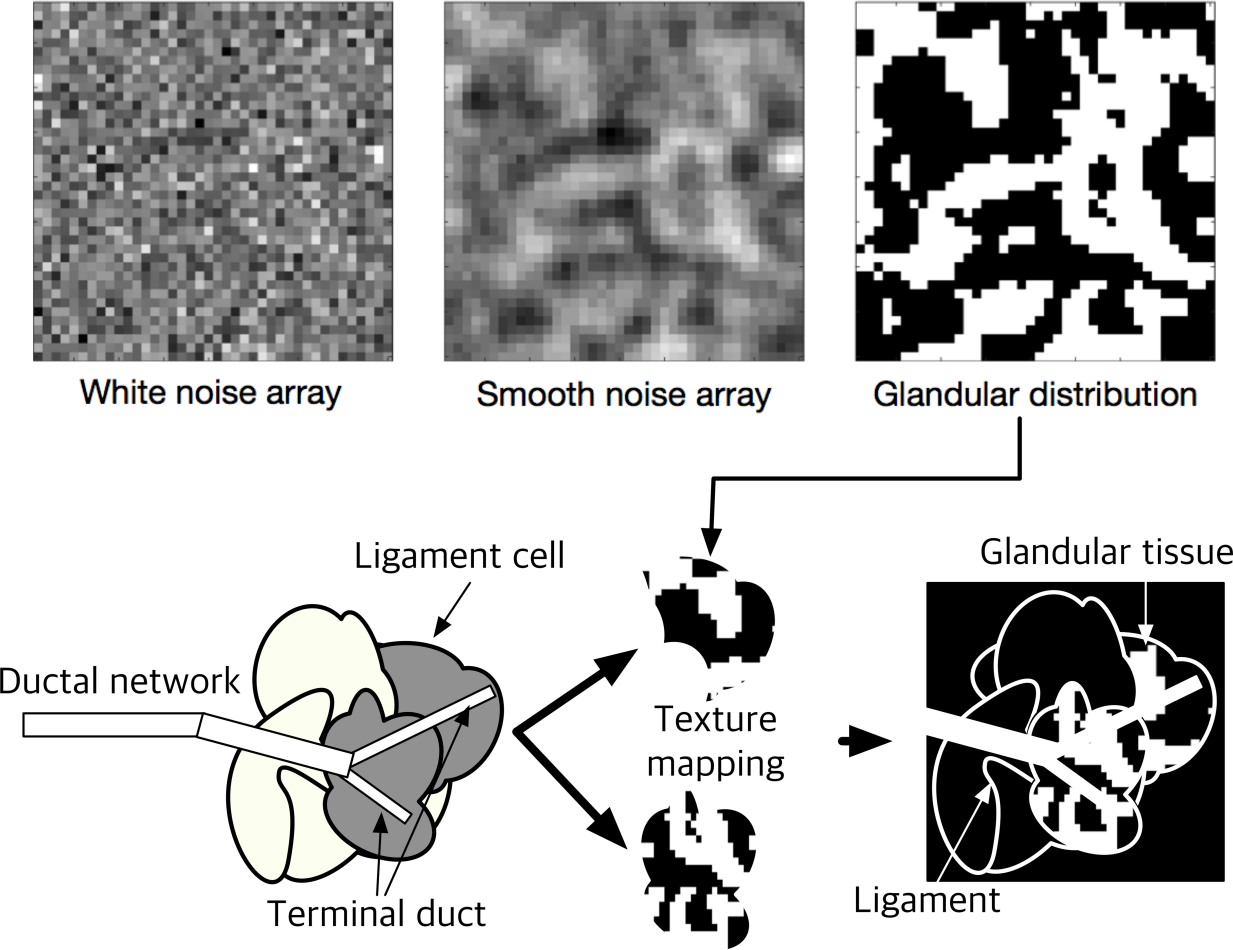
Generation of glandular tissue distribution.

Therefore, α was fixed in this study, but we adjusted the glandular tissue density by changing the threshold value, which is related to the array noise magnitude. We used the threshold value as the mean value for the entire volume. If the breast density can be controlled, then the threshold level can be adjusted as necessary to achieve a certain objective. An example of noisy-array filtration and thresholding is depicted in the upper panel of Fig 4.

After the ligament structure and lobular tissues are defined, the glandular tissue is constructed using the binary “AND” operation between the internal part of the ligament structure and lobular tissue. Based on anatomical knowledge, the lobules are located at the ends of the terminal ducts; therefore, the cells of the ligament set are categorized as “terminal part of ductal network was passed through” or “was not.” The terminal duct can be regarded as having the diameter of the ductal cylinder already simulated, which was recorded during ductal network generation. The marked ligament cells (cells categorized as “ductal network was passed through”) are eventually filled with the lobular tissue. A schematic illustration of the combination of the ductal network, Cooper’s ligament, and lobular tissue is provided in the lower panel of Fig 4.

#### Design of abnormal tissue

Numerical phantoms can have quantitative or qualitative abnormalities added. Abnormalities are intentionally added to determine the performance limits of systems in general. Thus, quantitative abnormalities can be bar patterns or contrast detail inserts made of geometric figures. Such inserts can easily be modeled for specific purposes, as should be done with abnormalities when utilizing this phantom. Therefore, we did not address abnormalities in this study.

### Polychromatic forward projection

The X-ray photon incidence on the detector surface after phantom attenuation *p*(*ε*) follows Beer’s law,

$$p(\epsilon )=\Phi_{0}(\epsilon )e^{\int -\mu (r,\epsilon )dl(r)},$$ (8)

where *μ*(*r*, ε) and *d*l(*r*) denote the energy-dependent total attenuation coefficient and interaction length along the ray, respectively, and Ф_0_(*ε*) is the incident spectrum of the number of photons generated at the X-ray source in terms of *ϵ*. For typical energy-integrating detectors, the numbers of generated signal carriers (i.e., optical photons and electron-hole pairs for indirect and direct conversion detectors, respectively) are proportional to the X-ray energy; hence, the signal value at a particular pixel *P* can be expressed as

$$P=\int_{\epsilon }\epsilon p(\epsilon )d\epsilon .$$ (9)

To simulate Eq 9 numerically, we first simulated Eq 8 for an entire pixel using the ray-driven algorithm proposed by Siddon, which is a typical forward projection algorithm [14]. Since the algorithm involves extensive computation, it is very inefficient to apply Eq 8 repeatedly for all of the energy bins in the spectrum. To overcome this issue, we used the concept of thickness projection. Similar attempts have been made in previous studies on beam-hardening artifact correction, and we have applied it to polychromatic projection simulations.

**Fig 5:**
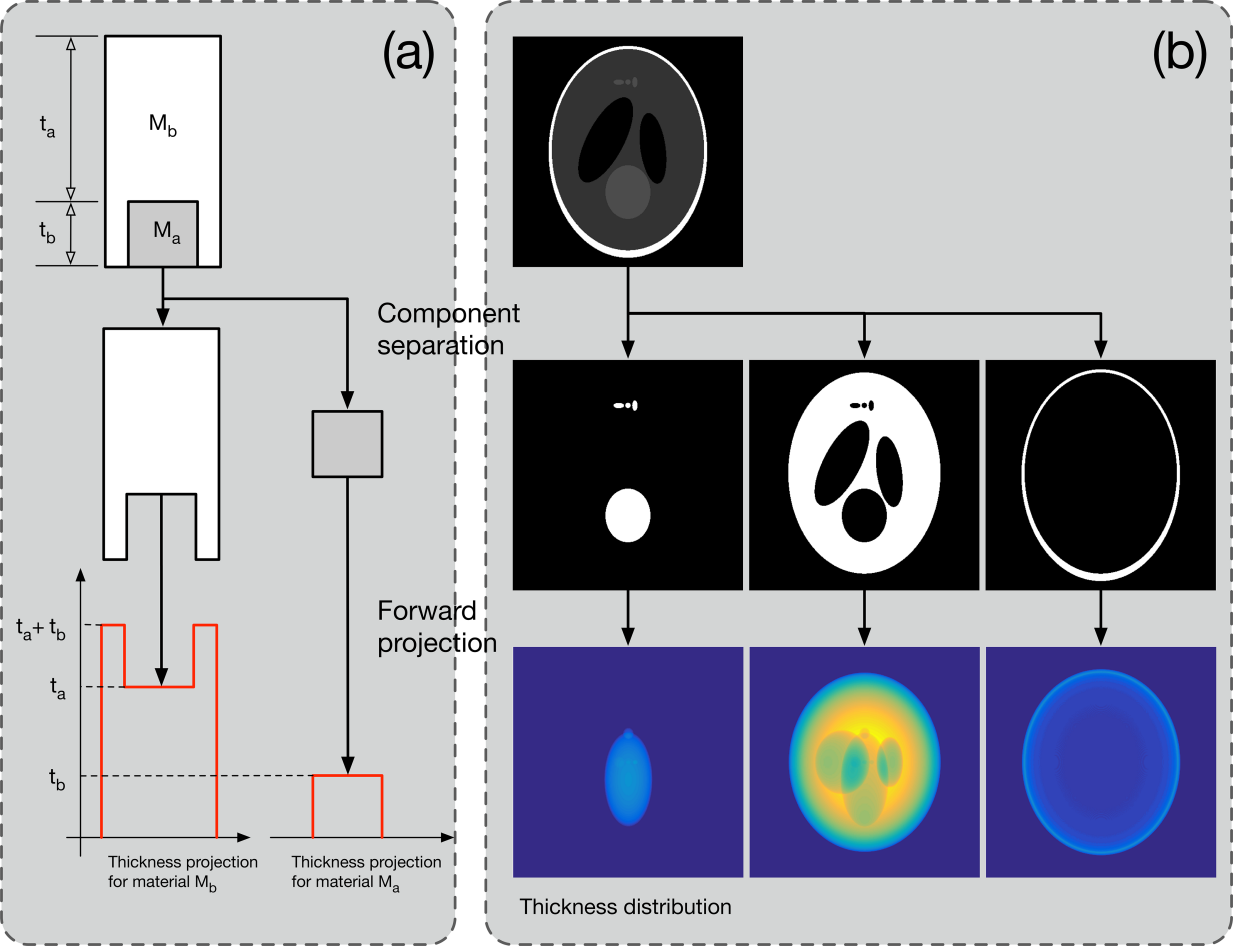
Conceptual illustration of the thickness projection calculations. A numerical phantom composed of two materials (*M*_*a*_ and *M*_*b*_) is first separated into two voxel phantoms. Then, ray tracing to each phantom is performed. The thickness projection contains only the spatial thickness distribution along the X-ray source and detector geometry.

A thickness projection is a virtual projection image that only contains the thickness distribution along each ray for certain phantom components [28, 29]. Therefore, if only the first forward-projected line is executed, arithmetic operations can be substituted for the subsequent energy. The thickness projection computation method is illustrated conceptually in Fig 5. Assuming that the phantom is composed of substances *M*_*a*_ and *M*_*b*_, it can be separated into individual phantoms containing only *M*_*a*_ or *M*_*b*_. Then, as shown in the figure, the thickness projection of each material can be calculated. Let us define the thickness projections as *P*_*t*,*Ma*_ and *P*_*t*,*Mb*_. If the energies of incident photons are *ε*_1_, *ε*_2_, and *ε*_3_ and the numbers of incident photons are Ф(*ε*_1_), Ф(*ε*_2_), and Ф(*ε*_3_), respectively, then the polychromatic projection can be defined as

$$P=\overset{3}{\underset{n=1}{\sum }}\epsilon_{n}\times e^{-[\mu (M_{a};\epsilon_{n})P_{t,M_{a}}+\mu (M_{b};\epsilon_{n})P_{t,M_{b}}]}.$$ (10)

Two steps are required to calculate *P*_*t*,*Ma*_ and *P*_*t*,*Mb*_ using the forward projection method, and the remaining operations are replaced with simple arithmetic operations. Although the energy of the incident photons is simplified to three values in this example, it is anticipated that considerable reduction in computational cost will be achieved if dozens of energy intervals exist in a general spectrum.

The X-ray spectrum was simulated using existing empirical models such as the tungsten and molybdenum anode spectral model interpolating polynomials libraries [30–32]. The mass attenuation coefficients of breast tissues were taken from NIST XCOM [33].

## Results

### Numerical breast phantom and its variation

**Fig 6:**
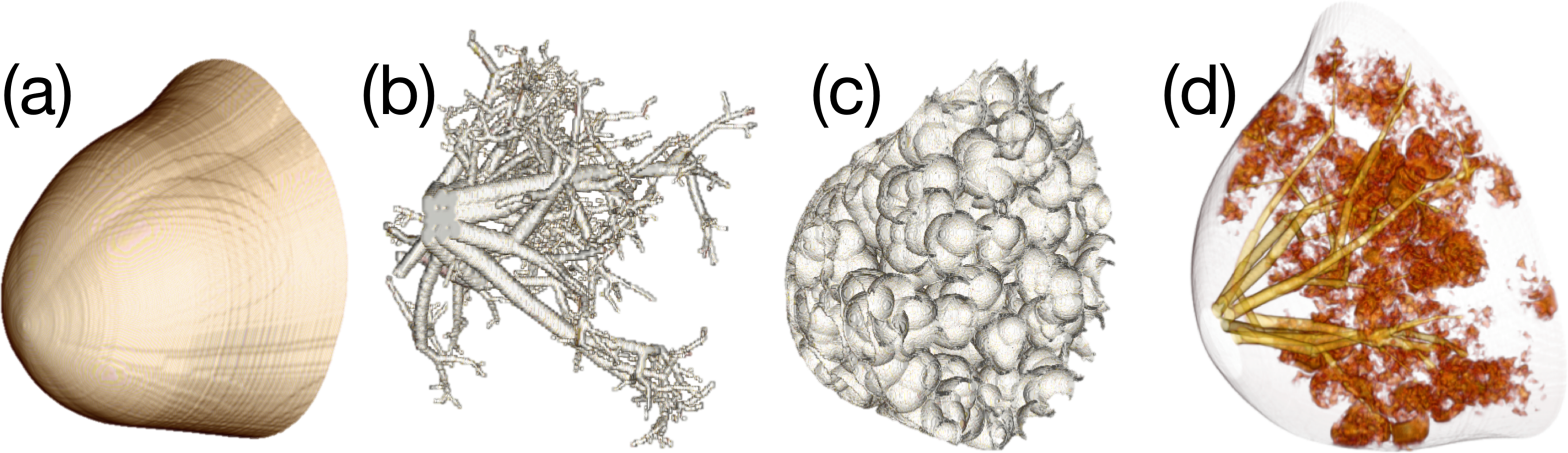
Modeled breast phantom compartments: (a) external boundary, (b) ductal network, (c) Cooper’s ligament, and (d) glandular tissue distribution.

Examples of the generated breast phantom compartments are shown in Fig 6, where Fig 6(A), 6(B), 6(C) and 6(D) are the 3D volume renderings of the skin, ductal network, Cooper’s ligament, and glandular tissue (lobule), respectively. Because the numerical phantom is generated randomly, its shape changes every time the computations are performed. In this study, the volume rendering was conducted using the commercial DICOM viewer (OsirixMD, Pixmeo SARL, Switzerland). The image of Cooper’s ligament in Fig 6(C) depicts the hollow membranes only. The voxel dimensions were 512 × 512 × 512 and the isotropic voxel size was 0.4 mm.

**Fig 7:**
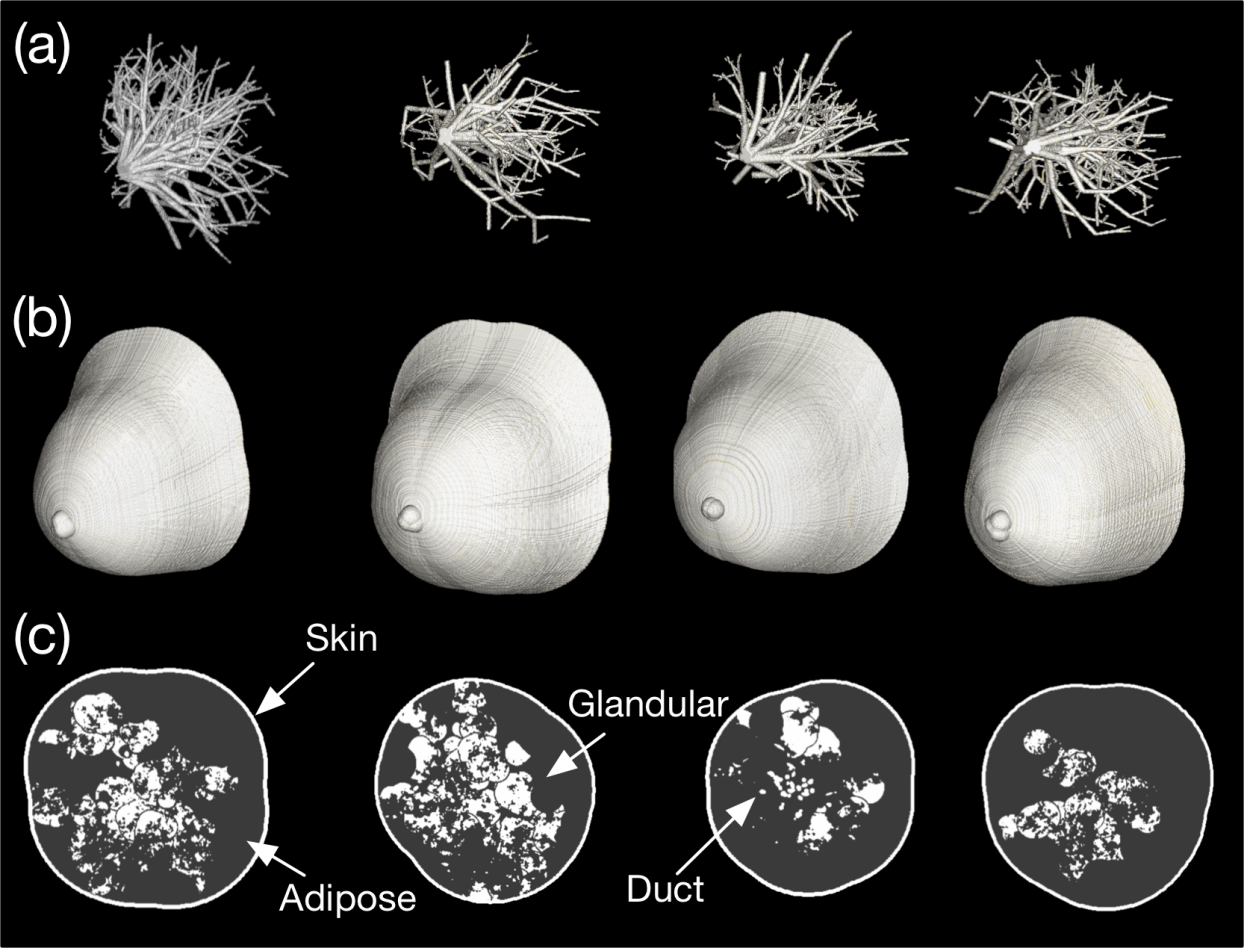
Volume renderings of the generated software breast phantoms: (a) ductal network, (b) external boundary, and (c) central slice views of the phantoms.

The breast phantoms generated in four attempts are presented in Fig 7, where Fig 7(A), 7(B) and 7(C) depict the ductal networks, external shapes, and central slices, respectively. All of the random variables were as listed in Table 1. Although we did not force the random variables, the breast density varied considerably, as shown in Fig 7(C). We assigned the linear attenuation coefficient of breast tissue to be 54 keV, which is the approximate mean energy of the tungsten anode spectrum for a BCT tube voltage of 80 kVp. The software phantom depicted in the first column of Fig 7 was published online as an example [34]. Integer values of 1, 2, 3, and 4 were assigned to adipose tissue, skin, glandular tissue, and Cooper’s ligament, respectively. The attenuation coefficient and density of each substance were not considered because they vary depending on the selected reference, purpose, and breast characteristics in actual applications. Furthermore, the Cooper’s ligament in Fig 7(C) was ignored because of the figure resolution. Cooper’s ligament can be indirectly supposed based on the distribution of glandular tissue, since Cooper’s ligaments surround the glandular and adipose tissue.

### Polychromatic projections

**Fig 8:**
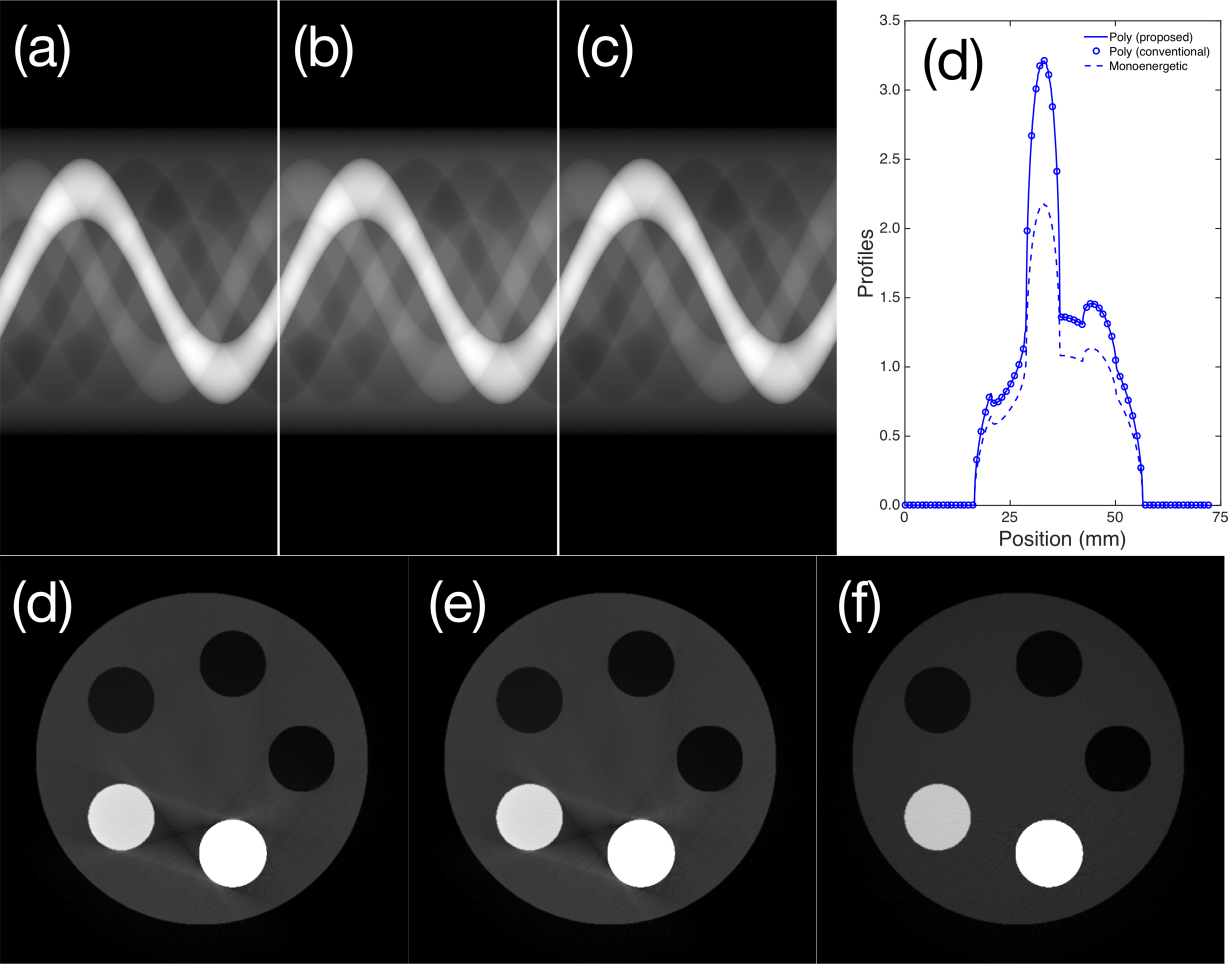
Sinograms acquired from the cylindrical phantom using the (a) proposed and (b) conventional polyenergetic sampling and (c) monoenergetic (35 keV) sampling. Profiles of the center of the sinogram: image reconstructions based on the (d) proposed and (e) conventional polyenergetic sampling and (f) the 35 keV monoenergetic sinogram.

To investigate polychromatic projections, we fabricated a simple cylindrical phantom with aluminum and Teflon inserts. We applied the total attenuation coefficient to the thickness projections to obtain the polyenergetic projection images in Fig 8(A) and 8(B) using the thickness-projection-based and energy bin repetition methods. The monoenergetic sinogram corresponding to 35 keV photon incidence is depicted in Fig 8(C). To facilitate visualization, the center profile of each sinogram is depicted in Fig 8(D). As shown in the profiles, the results of the proposed and conventional methods are identical. We compared the two projections pixel by pixel, and the overall error was estimated to be exactly 0%. However, the monoenergetic sinogram had an average relative error of 15.2%. Note that the log transform was incorporated into the raw sinograms and profiles to improve the image visibility. We determined the relative error *ι*_*r*_ using

$$\iota_{r}=\frac{\left|I_{poly}-I_{mono}\right|}{I_{poly}}\times 100.$$ (11)

The images reconstructed based on the polyenergetic and monoenergetic spectra are depicted in Fig 8(E) and 8(F), respectively. The beam hardening artifacts arising between dense materials (aluminum and Teflon inserts in this case) are clearly observable. The results obtained with proposed and conventional polyenergetic sampling were confirmed again. As expected, beam hardening artifacts are not apparent in the monoenergetic image shown in Fig 8(F). The gray value distributions also differ slightly between the monoenergetic and polyenergetic results. An in-house filtered back-projection algorithm with fan-beam geometry was applied using the Hanning apodization filter [35].

**Fig 9:**
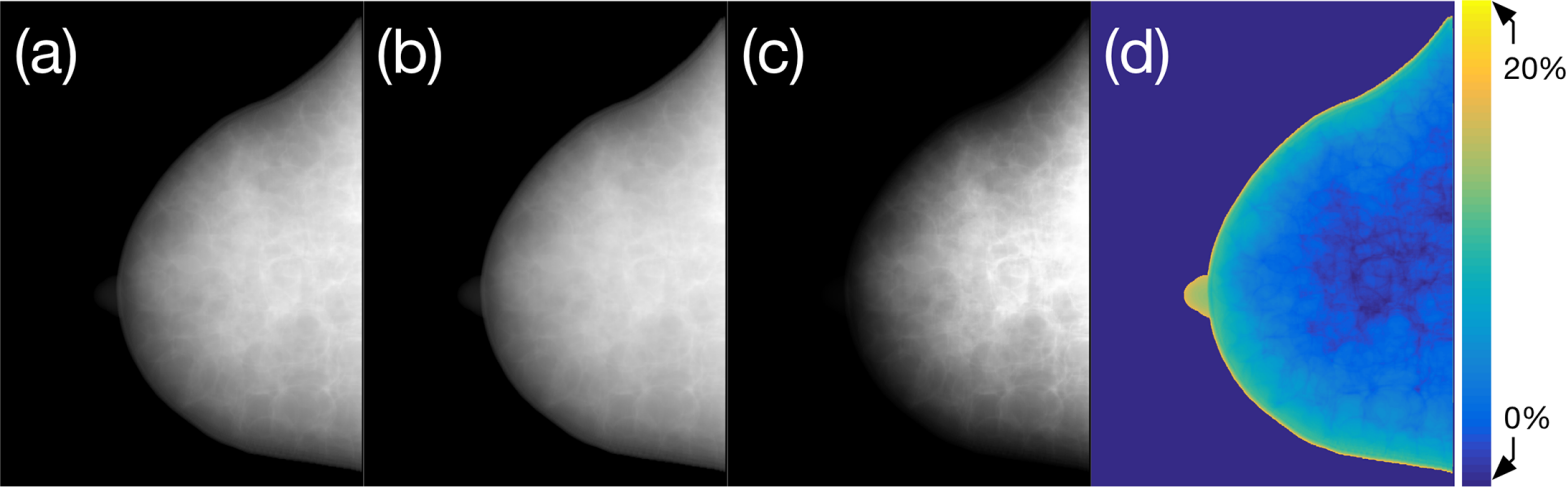
Comparison of the monochromatic and polychromatic projection images of the breast phantom: (a) monochromatic projection image for 35 keV photon incidence, which is the mean energy of the 60 kVp tungsten anode spectrum without additional filtration; (b) polychromatic projection of the 60 kVp spectral photon incidence; (c) histogram-equalized image of (b) to facilitate visualization; and (d) relative error between (a) and (b) as a percentage.

Projection images acquired using monochromatic and polychromatic spectrum incidence are presented in Fig 9 for the breast phantom depicted in the first column of Fig 7. The monochromatic and polychromatic projection images obtained with a 35 keV and 60 kVp tungsten anode spectrum and without additional filtration are shown in Fig 9(A) and 9(B), respectively. The mean energy of the 60 kVp spectrum, 35 keV, is widely used in monochromatic assumptions for forward-projection-based image simulations. The histogram-equalized output of the polychromatic projection is provided in Fig 9(C) to facilitate visualization of the internal structure distribution. The log transform was taken to obtain Fig 9(A)–9(C), and Fig 9(D) is a map of the relative error between Fig 9(A) and 9(B).

The maximum relative error was determined to be approximately greater than 20% at the nipple and edge of the breast boundary. The region mainly composed of adipose tissue had a higher error than the glandular-tissue-dominant regions. We assumed that the source-to-detector and source-to-rotation axis distances were 700 mm and 500 mm, respectively, based on a previous publication [36]. The imaging detector was assumed to have 1024 × 1024 pixels with a pixel size of 0.3 mm × 0.3 mm; therefore, the active area of the flat-panel detector was 307.2 mm × 307.2 mm. Since we cut off the marginal area of the detector, the projection images depicted in figure are not square.

**Fig 10:**
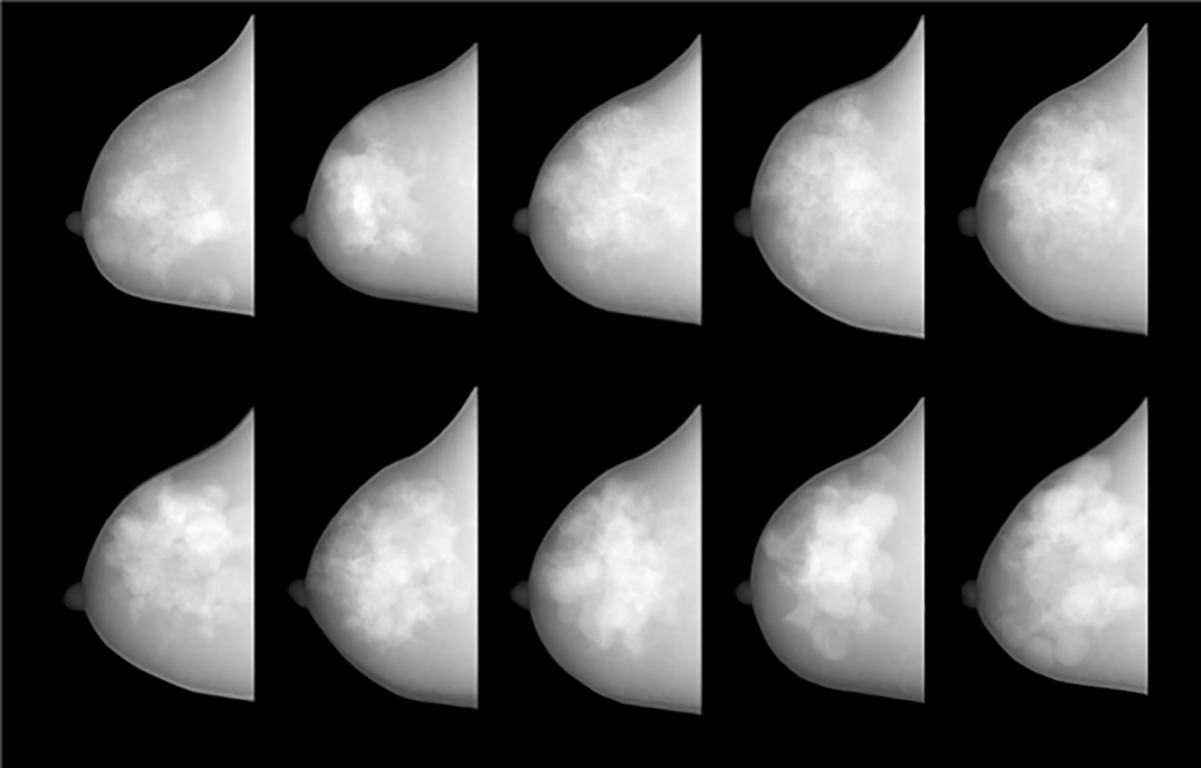
Lateral projection images simulated using breast phantoms of various densities: Glandular tissue comprises 7.6%, 11.8%, 12.4%, 16.5%, 16.8%, 22.1%, 22.4%, 22.8%, 23.5%, and 27.1% of the total volume (clockwise from the upper left corner).

The polychromatic X-ray projection images obtained using random breast phantoms generated with the same parameter sets as those employed to produce the phantom depicted in Fig 8 are presented in Fig 10. The scanner geometry and specifications of the imaging detector were the same as those utilized to obtain Fig 9. Since the type of software phantom proposed in this report can be randomly generated, it is possible to obtain a differently shaped phantom each time a phantom is produced. The internal configuration of the phantom, such as the glandular and adipose tissue distribution, changes with each attempt; therefore, adipose to glandular ratio also changes. The projection images of the computational phantoms generated in 10 attempts are also depicted in Fig 10. Because we defined basic constraints for the external and internal structures, the shapes are roughly similar, although the details differ. The glandular tissue comprises 7.6%, 11.8%, 12.4%, 16.5%, 16.8%, 22.1%, 22.4%, 22.8%, 23.5%, and 27.1% of the total volume of each phantom, clockwise from the upper left corner.

In this study, we did not adjust the basic parameters to control the glandular tissue ratio or distribution. Therefore, the phantom trends shown in the Fig 10. are due to purely random behavior. Of course, the physical parameters of the phantom can be changed considerably by adjusting the basic parameters, especially the lobular tissue threshold, number of lobes, and external contour diameter. However, these adjustments were not within the scope of this study because they should be designed according to the purposes of specific applications of this type of software phantom.

## Discussion and conclusion

We developed a forward-projection-based simulation method to mimic dedicated BCT systems that can describe a realistic uncompressed breast phantom with a polyenergetic X-ray spectrum. Unlike in previous studies, the breast phantom was generated using random variables with an uncompressed breast suitable for dedicated BCT. The concept of polyenergetic sampling is not novel, but the proposed computational reduction method is useful for making polyenergetic sampling practical. Because the proposed algorithm only reduces the number of ray tracings as a factor of the ratio between the energy bins and number of components, any accelerated ray-tracing algorithm, such as the distance- and ray-drive algorithms, can be applied. In addition, the calculations can be accelerated using a GPU.

Because the programming code developed in this study was not optimized, the absolute computation time is not meaningful. However, if the calculation code is optimized and parallelization using a GPU or equivalent hardware is applied, the speed is expected to increase by a factor of approximately 10. Furthermore, Matlab, which was used to implement the algorithm, is a script language. Therefore, the computations are inherently slow compared to those performed using compiled languages such as C++, Compute Unified Device Architecture (CUDA, Nvidia Co., CA, USA), and OpenCL [37].

In addition, we did not consider abnormalities in the phantom and virtual projections in this study. Abnormalities, especially micro-calcifications and masses, are the most important factors in breast cancer diagnosis. In general, micro-calcifications can be assumed to be finely scattered high-contrast media (e.g., CaCO_3_), and masses can be modeled as low-contrast spheres. However, abnormalities need to be modeled considering their occurrence mechanisms through more intensive further studies. Because abnormality diagnosis is the ultimate goal of dedicated BCT, poorly designed abnormalities can cause fatal errors throughout the phantom and simulation process. At the present stage, however, it is possible to replace factual abnormalities by inserting quantitative abnormalities assumed to be star patterns or spheres into the phantom. In future research, we will add abnormalities to the phantom based on the disease mechanism and publish the results.

In summary, the proposed simulation method will be effective for use in computer simulations to predict and/or compare the performances of radiological imaging systems, especially dedicated BCT systems. Thus, it could be very helpful for imaging-system designers and engineers. In addition, it will be useful for medical physicists since it will facilitate the optimization of image acquisition parameters for almost all labor-intensive experimental research [38]. We also expect this method to be capable of replacing Monte Carlo simulations in simple cases by adding quantum noise to the projected images calculated using this algorithm. Although monoenergetic forward projection is used in the iterative image reconstruction algorithm, polychromatic forward projection model could be applicable to the projection model of the model-based iterative reconstruction algorithm, which has recently gained attention [39, 40].

This study was focused on acquiring realistic projection images that can be used for the early-stage development of dedicated BCT systems without requiring experiments to be conducted. In particular, the projection images thus obtained will greatly facilitate studies of image reconstruction and pre/post-processing algorithms. Although this study was intended to enable the production of realistic phantoms and projection images, it is not possible to achieve sufficient realism to cause radiologists to misjudge true breast images. However, since Shepp–Logan head phantoms, which are widely used in this type of research, are too simplistic to overestimate the performances of algorithms or systems when using phantoms capable of realistically simulating human bodies, this study can be a useful tool to predict the upper performance limits of the systems and algorithms being developed.
